# Section’s osseous slice biopsy during major amputation of lower extremity: preliminary results of prospective cohort study

**DOI:** 10.1186/s12879-015-0993-x

**Published:** 2015-06-30

**Authors:** Danguole Vaznaisiene, Rita Sulcaite, Astra Vitkauskiene, Arturas Spucis, Anatolijus Reingardas, Vytautas Kymantas, Kestutis Balanaska, Rolandas Sleivys, Linas Velicka, Juozas Belickas, Kristina Rysevaite-Kyguoliene, Dainius H. Pauza, Aukse Mickiene, Eric Senneville

**Affiliations:** Department of Infectious Diseases, Lithuanian University of Health Sciences, Kaunas, Lithuania; Institute of Endocrinology, Lithuanian University of Health Sciences, Kaunas, Lithuania; Department of Laboratory Medicine, Lithuanian University of Health Sciences, Kaunas, Lithuania; Department of Surgical Infection, Republican Hospital of Kaunas, Kaunas, Lithuania; Department of Surgery, Lithuanian University of Health Sciences, Kaunas, Lithuania; Department of Surgery, Kaunas Clinical Hospital, Kaunas, Lithuania; Department of Cardiothoracic and Vascular Surgery, Lithuanian University of Health Sciences, Kaunas, Lithuania; Department of Orthopedics and Traumatology, Lithuanian University of Health Sciences, Kaunas, Lithuania; Institute of Anatomy, Lithuanian University of Health Sciences, Kaunas, Lithuania; Infectious Diseases Department, Dron Hospital, Tourcoing, France

**Keywords:** Bone biopsy, Major amputation, Osteoarticular infections

## Abstract

**Background:**

The purpose of this cohort study was to assess the incidence of positive cultures in section’s osseous slice biopsy (SOB) taken at the level of major limb amputation. In case of positive cultures we sought whether the microorganisms present in SOB could take origin from the primary infection site necessitating the amputation. The impact of diabetes on culture results was also investigated.

**Methods:**

This prospective cohort study, which aimed to confirm the results of the pilot study, analysed patients who underwent major limb amputation between 2012 and 2013 in three Lithuanian hospitals. SOBs at the amputation site (surgical bone biopsies) and percutaneous bone biopsies of the distal site were performed simultaneously during limb amputation. Tissue cultures were analysed by microbiologists, and species along with antibiograms were reported. Histopathological assessment and bacterial typing were also evaluated. A positive culture was defined as the identification of at least 1 bacteria not belonging to the skin flora, at least 2 bacteria belonging to the skin flora with the same antibiotic susceptibility profiles or the same bacteria belonging to the skin flora in two different sites. Fisher’s exact test and Student’s test were used to compare the populations and the microbiological results. The statistical significance level was set at *P* < 0.05.

**Results:**

Sixty-nine patients (35 males/34 females), mean age 68.7 (S = 13.6) years, including 21 (30.4 %) with diabetes underwent the major limb amputation. Forty-five amputations (65.2 %) were done above the knee. In total, 207 SOBs and 207 percutaneous distal site biopsies were studied. SOB cultures were positive in 11 (15.9 %) cases. In 5 (45.5 %) cases the same microorganisms were identified in both SOB and distal biopsy cultures. No association between culture results and presence of diabetes was identified.

**Conclusions:**

Our results suggest that, independently of the diabetes status, foot infection may silently spread along the bone and can achieve the site of major limb amputation. Additional investigations aiming to confirm this hypothesis and to evaluate a prognostic value are in progress.

## Background

Section’s osseous slice biopsy (SOB) is rarely performed in routine amputations, partially due to additional cost and the threat of increased risk of adverse events. However, the belief that the proximal amputation site distant from the site necessitating the limb amputation is unlikely to be involved in the infectious process, is still frequent and this may explain why SOB is regularly omitted from the amputation site selection process. Data about the probability of the extension of the infectious process affecting the distal site responsible for the amputation, prognostic value are lacking and findings present have only been established by taking superficial microbiology cultures.

We hypothesized that infection from the distal site may spread along the bone and be present at the amputation site. Therefore, a positive SOB culture could be a risk factor for delayed stump healing. Our team has previously participated in a retrospective pilot study in France, which compared the culture results of SOB with those of bone biopsy at the initial distal infection site [1]. The results of this study suggested that SOB cultures are often positive even if the level of limb amputation is distant from the infection source responsible for the amputation, and that a positive culture of the SOB is a risk factor for delayed stump healing. However, the study was limited by retrospective design, small population size and failure to use histopathological assessment for the diagnosis of osteomyelitis. To confirm the findings from this previous study we decided to conduct a prospective cohort study in institutions where SOB or percutaneous bone biopsies are not routinely performed during the amputation.

## Methods

This is a 1 year follow-up cohort study. Stump wound healing, postoperative antibiotic therapy will be assessed at the secondary end point of the study.

In this preliminary study we report the incidence of positive cultures in section’s osseous slice biopsy (SOB) taken at the level of major limb amputation and compare the microbiology of positive cultures with percutaneous bone biopsy cultures performed at distal infected site. We also analyze the relationship between diabetes and microbiological culture results.

### Population

In this multicenter prospective cohort study we included adult patients who underwent a major amputation of lower limb in the Hospital of Lithuanian University of Health Sciences Kauno klinikos, Kaunas Clinical Hospital and Republican Hospital of Kaunas between 2012 and 2013. The only exclusion criterion was the refusal to participate in the study. Patients had a complete medical history taken, full examination and tests performed. The following data were collected: age, sex, diabetes status and glycemic control, presence of peripheral vascular disease, osteoarthritis, level of infection, context of trauma, level of amputation, immunosuppression, bedridden disability, comorbidity, duration and history of the pathology, reason of the amputation, time from the decision to amputate to surgical procedure, history of previous amputations, total duration of antibiotic therapy before the amputation, antibiotic-free interval before amputation, inflammatory markers (C-reactive protein), renal function (creatinine, estimated glomerular filtration rate), and antibiotic use before and after hospitalization.

Peripheral vascular disease was assumed to be present if 1) dorsal-pedal and posterior-tibial pulses were absent and/or 2) pathological results were evident on Doppler UltraSound, peripheral arteriography and/or 3) there was a previously made diagnosis of peripheral vascular disease by vascular surgeon by using the methods mentioned above. Comorbidity status was evaluated on the presence of cardiac, renal or hepatic insufficiency. Patients were considered to have had an antibiotic-free interval, if they had not received any systemic antibiotic for at least 2 weeks before the amputation. An informed consent form had to be completed by every patient who met the inclusion criteria. The study protocol was approved by Lithuanian Ethics Committee (Number BE-2-23, 2012 May 14).

### Specimen collection

Concomitant SOB and percutaneous bone biopsy of the distal site were performed during limb amputation. A new surgical setup and new instruments were used, to try and decrease the likelihood of cross-contamination during surgery. Percutaneous bone biopsy was performed in the surgical room using an 11-gauge biopsy needle inserted through a 5–10-mm skin incision at least 20 mm from the ulcer periphery, to avoid contamination by the colonizing flora, following the methodology described in the literature [2]. During the amputation, in the surgical room we performed SOB at the level of major limb amputation from the amputated limb part using Liston bone cutting forceps and removing the bone fragments (cortical bone and bone marrow for each biopsy). Three bone fragments were obtained for both SOBs and percutaneous distal biopsies, two of which underwent the microbiological assessment, the rest bone fragment - histopathological assessment.

### Microbiological assessment

Specimens were immediately placed into Amies transport medium (Brescia, Italy), brought to the microbiology laboratory within 1 h after sampling. The samples were inoculated directly into 5 % sheep blood agar (BBL, USA), chocolate agar (BBL, USA), Mac Conkey agar plates (Oxoid, UK), Schaedler agar (BBL, USA) and thioglycollate broth. Sheep blood and chocolate agar plates were incubated at 35 °C in an atmosphere containing 5 % CO_2_ and Mac Conkey agar plates – at 35 °C for 18–24 h. If culture was negative at the first observation, sheep blood and chocolate agar plates were re-examined after a second 24 h incubation. Thioglycollate broth was incubated at 35 °C temperature for 5 days. Schaedler agar was incubated at 35 °C in an anaerobic workstation Bug Box (UK) for 2 weeks. Maldi-TOF-MS (BRUKER) mass spectrometry was used for microorganism identification. Analysis of the number of colony-forming units per culture was performed by means of a counting frame for bone biopsy cultures. A positive culture was defined as the identification of at least 1 bacteria not belonging to the skin flora, at least 2 bacteria belonging to the skin flora (CoNS (coagulase negative staphylococci), *Corynebacterium spp*) with the same antibiotic susceptibility profiles or the same bacteria belonging to the skin flora in two different sites. A doubtful culture was defined as the identification of one bacteria belonging to the skin flora in one site. The dominant pathogen required at least a three times higher growth in bone cultures than other microorganisms. All positive cultures were frozen at −60 °C for further research. The isolated pathogens from SOB and distal site biopsy, which happened to be the same species were compared using genotyping (pulsed field gel electrophoresis) to confirm the same strain.

### Histopathological assessment

Bone fragment was fixed in 4 % paraformaldehyde, decalcified in 10 % HCl and embedded in paraffin for histological analysis. 3-μm thick sections were made, stained with hematoxylin-eosin.

Histopathological findings for the signs of infection were evaluated. Histopathological findings in bone specimens were defined as acute osteomyelitis when necrosis, destroyed bone and infiltrations of polymorphonuclear granulocytes at cortical sites and inside the bone marrow were present. Congestion or thrombosis of medullary or periosteal small vessels was also a frequent finding. It was defined as chronic osteomyelitis when there was destroyed bone and infiltrations of lymphocytes, histiocytes and/or plasmatic cells at cortical sites and inside the bone marrow. All cases of osteomyelitis exhibited areas of fibrosis in variable forms and medullar oedema.

### Statistical analyses

Fisher’s exact test and Student’s test were used to compare the populations and microbiological results, all the variables had normal distribution. The statistical significance level was set at *P* < 0.05.

## Results

**Fig. 1:**
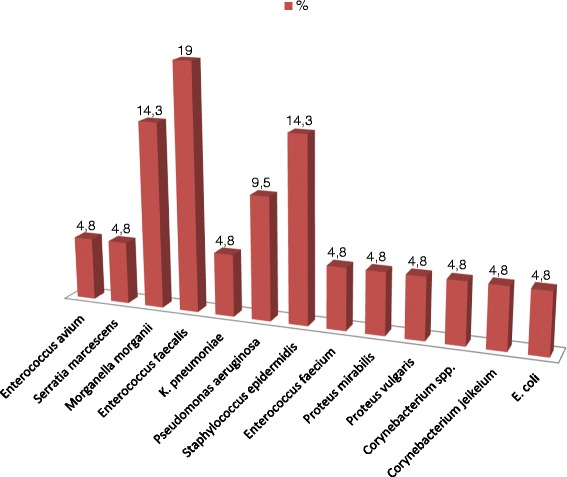
Microorganisms isolated from SOB

From 2012 to 2013, 69 patients (35 males/34 females, age 68.7 (S = 13.6)) underwent major limb amputation at Kaunas hospitals. Among all these 69 patients, 45 (65.2 %) had above the knee amputation and 21 (30.4 %) were known to have diabetes (Table 1). 207 SOBs at amputation level and 207 distal percutaneous biopsies were taken, and 138 of each underwent the microbiological assessment, 69 of each – histopathological assessment. In 17 cases (24.6 %) the microorganisms were identified in SOB. SOB cultures were evaluated as positive in 11 (15.9 %) cases (Fig. 1). 83.3 % Enterococcus isolates were sensitive to vancomycin. Susceptibility of Gram-negative organisms to ciprofloxacin was 87.5 %. The rate of resistance of Pseudomonas aeruginosa to imipenem and ceftazidime was 50 % for each. In 2 cases the signs of infection were found in histopathological assessement of SOB.

**Table 1:** Population and the results of bone biopsies

	Variable	Prospective cohort study (n = 69)
Population	Mean age	68.7 (S = 13.6)
	Male	35 (50.7 %)
	Amputation above the knee	45 (65.2 %)
	Of them due to peripheral vascular disease	40/45 (88.9 %)
	Diabetics	21 (30.4 %)
	Diabetic foot	14 (20.3 %)
	Trophic disorders	52 (75.4 %)
	Trauma	24 (34.8 %)
	Antibiotic-free interval before limb amputation	39 (56.5 %)
SOB	Sterile SOB	52 (75.4 %)
	Positive SOB	11 (15.9 %)
	Doubful SOB	6 (8.7 %)
	Positive + doubtful SOB	17 (24.6 %)
	No. of isolates	27
	No. of isolates, by pathogen	
	*S. aureus*	0
	Coagulase-negative staphylococci (in all)	9 (33.3 %)
	*Staphylococcus epidermidis*	7
	*Staphylococcus warneri*	1
	*Staphylococcus cohnii*	1
	Other gram- positive bacteria	8 (29.6 %)
	Gram-negative bacilli	10 (37 %)
	Anaerobes	0
	Methicillin resistant staphylococci	3/9
SOB and percutaneous bone biopsy of the distal site	Microorganisms identified from SOB were found in the distal infected site	45.5 %
	*S. aureus*	0
	Coagulase- negative staphylococci	1/9
	Other gram- positive bacteria	2/7
	Gram-negative bacilli	3/10
	Anaerobes	0
	Methicillin resistant staphylococci	0/3

**Fig. 2:**
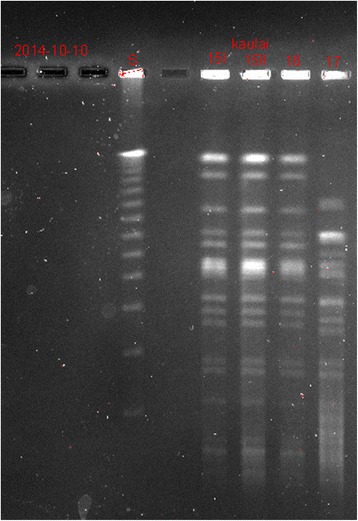
Species comparison using genotyping (pulsed field gel electrophoresis) to confirm the same strain

In 5 (45.5 %) cases the same microorganisms (same species, antibiogram, genotype) were identified in SOB and distal site bone biopsy cultures (Fig. 2). No species were found to be particularly predominant (Table 2).

**Table 2:** Species of matching microorganisms isolated from both SOB and percutaneous distal infected bone biopsy cultures

Microorganism	Number of patients
*Morganella morganii*	1
*Enterococcus faecium*	1
*Pseudomonas aeruginosa, Staphylococcus epidermidis*	1
*Proteus mirabilis*	1
*Enterococcus faecalis*	1

SOB cultures were positive in 5 (23.8 %) patients suffering from diabetes and in 6 (12.5 %) patients without diabetes (p = 0.29). Microorganisms identified in SOB were also cultured from the distal site in 1 (20 %) diabetic and 4 (66.7 %) non-diabetics (p = 0.24) (Table 3).

**Table 3:** Comparison of the bone biopsy results in diabetics and non-diabetics

	Diabetics (n = 21)	Non-diabetics (n = 48)	P
Positive SOB	5 (23.8 %)	6 (12.5 %)	0.29
Microorganisms identified from SOB were found in the distal infected site	1/5 (20 %)	4/6 (66.7 %)	0.24

## Discussion

Studies comparing the microbiological cultures of SOB and the cultures from the site necessitating amputation are lacking. Previously reviewed reports on how infectious processes influence the amputation outcome have, however, been established using the superficial samples [1]. Taking superficial samples for the diagnosis of osteomyelitis of diabetic foot, which is a widespread practice, is of limited or at least uncertain diagnostic value [2, 3]. In case of non-diabetic bone infection (including stump infections) the swab bacterial cultures are often misleading, and bone biopsy remains the optimal management as well [4, 5]. Bone biopsy is particularly valuable in providing reliable data on infecting organisms and their antimicrobial susceptibility [6].

In the retrospective pilot study carried out in France, the positive SOB cultures were more common than in our present study (p = 0.01). SOB cultures were positive in 42.1 % of the pilot study patients, while the microorganism at amputation and distal sites coincided in 69.6 % of the cases [1]. These discrepancies may be partially explained by a more radical surgery performed in Lithuania, as suggested by a greater percentage of the above-the-knee amputations as well as by a higher proportion of patients with peripheral vascular disease in our cohort. Antibiotic-free interval was observed in about a half of the cases in both cohorts. In the pilot study conducted at the National Reference Center as having complex osteo-articular infections, patients underwent routine SOB during major lower extremity amputation. In agreement with the results of the pilot study, we did not identified any difference between microbiological cultures obtained from diabetic and non-diabetic patients [1]. To the best of our knowledge, there are no similar studies reported in the literature.

In previous studies, the rate CoNS isolation from bone tissue varied between 10 % and 50 %, and was considered in most cases as true infections [2, 7, 8]. Lavery et al. [7] recorded that the intraoperative bone cultures used by their team were less prone to contamination than the percutaneous technique, and accounted for the lower CoNS incidence than in Newman series (11 % vs 50 %) [8]. In our study a chance of contamination remains high in spite of surgical bone biopsy (SOB). Three out of nine CoNS were evaluated as positive in SOB. Eight out of nine CoNS were not identified at the distal biopsy. Interestingly, one study found a higher rate of CoNS in the percutaneous bone biopsy samples than in the swab samples (25.6 % vs. 4.6 %; *p* < 0.001) [2]. This finding may support the idea that CoNS are the causative pathogens in these cases. However, the validity of this finding is unclear, because histological confirmation of osteomyelitis was not done [2]. Nevertheless, Aragon-Sanchez *et al.* presented a series of cases in which *S. epidermidis* was isolated from the bone biopsy and the histopathological studies, confirming diagnosis of osteomyelitis, but no anaerobic cultures were performed [9]. In the present study, the CoNS were the most common microorganisms grown from SOB (33.3 %), which corresponds to the literature data. In terms of CoNS abundance, the results of our previous pilot study did not differ from the present study, although in the pilot study all bone biopsies were performed by an experienced orthopedic surgeon, and the possibility of contamination was small. The other identified microorganisms did not differ significantly in both present and pilot studies.

Differentiating the true coagulase-negative staphylococcal infection from contamination has an important impact on therapeutic implications. In this situation, clinical, radiological findings, as well as histological signs of infection may be useful. In our study only in 2 cases the signs of infection were found in histopathological assessement of SOB. We think that the microorganisms spread and were found in SOB earlier until the changes are visible in histopathological or radiological analysis. Repeated positive cultures, isolation of identical species with comparable antibiotic susceptibility profile were considered as strong arguments in favour of a true infection in recent study [10]. In this study genotyping techniques demonstrated that CoNS exhibiting comparable antibiotic susceptibility patterns had a 93.3 % probability to belong to the same strain [10]. In our study in all cases isolated pathogens from SOB and distal site biopsy, which happened to be the same species and antibiogram were compared using genotyping and confirmed the same strain. In Bernard *et al.* study with quantitative cultures, coagulase negative staphylococci were considered as commensals, since their quantification was inferior to other concomitant and more classical pathogens of osteomyelitis [11]. Of note, genetic markers of virulence are unlikely to differentiate invasive and commensal strains [12–14].

To confirm our hypothesis that culture results of the slice site are reliable and bacteria ascend from the foot instead of from skin, blood, or other nearby source of infection and affect the amputation outcome, further investigations and analysis are in progress.

## Conclusions

Our results suggest that, independently of the diabetes status, foot infection may silently spread along the bone and can achieve the site of major limb amputation. Additional investigations aiming to confirm this hypothesis and to evaluate a prognostic value are in progress.
